# Bibliographical

**Published:** 1887-01

**Authors:** 


### BIBLIOGRAPHICAL.

A PRACTICAL TREATISE ON MECHANICAL DENTISTRY. By JOS. RICHARDSON, M. D., D. D. S. Fourth Edition: Revised and Enlarged, with four hundred and fifty-eight illustrations. Philadelphia: P. Blakiston, Son & Co. 1886.

For more than a quarter of a century Richardson's Mechanical Dentistry has been the standard text-book in its appropriate field.

More than one generation of dentists has drawn wisdom from its study, and many disputed points in mechanics have been decided by a reference to this acknowledged authority. But mechanical dentistry has made great advances of late, and a text-book that gives only the methods of but a very few years since is antiquated. Hence arose the necessity for a thorough revision of Richardson, and the work, as evidenced by the fourth edition, appears to have been well done. Since the earlier editions were issued entirely new methods of inserting artificial teeth have been devised, and the scope of dental mechanics has been immensely extended. It would be quite impossible in a book of this magnitude to present all the details of all the different kinds of work that have been proposed, but the author has, with rare good judgment, selected the methods which give the best results, and described them with sufficient exactness to serve as a guide to any intelligent man. In crown-work, for example, nearly thirty different methods are given. This does not cover all that has been devised, for the modifications are almost endless, but it includes all the principles involved, and is surely sufficient.

We are glad that the author persists in the use of the old and expressive term "Mechanical" in contradistinction to that modern affectation "Prosthetic." It savors of the day when a dentist was obliged to be a thorough *mechanic* in order to succeed; when the modern professional dilettanteism of the prosthetic dentist, who puts his cases into the hands of a skilled mechanic for the accomplishment of the work which he himself is too often incompetent to do, was a thing unheard of, and every capable dentist was competent to pursue every part of his profession.

We can commend the new edition of Richardson as the most complete work of the kind now before the profession.

Index to the Periodical Literature of Dental Science and Art, as Presented in the English Language. By J. Taft, M. D., D. D. S. Philadelphia: P. Blakiston, Son & Co. 1886.

Dr. Taft has, for all his active life, been identified with our professional literature, and there is, perhaps, no one who is better qualified to prepare an index to the periodical literature of dentistry than is he. At this day the most valuable essays upon subjects connected with dental practice are published in our journals, and he who depends solely upon text-books for his knowledge will not be thoroughly informed. Yet the articles upon any given subject are so buried up among many others that it has hitherto been impossible to collect the literature when its examination was desired. For years the editor of this journal has kept an *Index Rerum,* but has found it quite out of the question to note all that is of import. The work under notice is intended to supply the long-felt want of an index to our periodical literature, and it will prove of great benefit to any dentist who will study it. It is not absolutely complete, and there are some papers which we think should have been noted, but for which we have searched in vain. This may, however, be the result of a rather arbitrary system of indexing the titles. Sometimes the heads of articles are not sufficiently expressive of their scope, and sometimes they are much too long for insertion entire. In this case the titles must either be somewhat changed or abridged, and it is in these changes that confusion may, at times, be detected.

There are other papers indexed which we think scarcely worthy a place in our permanent literature—papers which contain much that is false and more that is foolish. But it would be difficult to so draw the line as to meet all objections, and the work, as a whole, will prove indispensable to every one who is interested in dental literature.

The Dental Review. Published Monthly for the Dental Review Company, by W. T. Keener, 96 Washington St., Chicago, Ill. $2.50 per annum.

We are in receipt of the initial number of the new dental journal announced in our last issue. It is a handsomely printed magazine of 56 pages, and bids fair to occupy a prominent place in our literature. The leading article is an illustrated paper, having for its title "The Periosteum and Peridental Membrane," by G. V. Black, M. D., D. D. S. Dr. Black is well known as one of our best original investigators, and his paper is an important contribution to our professional knowledge. It should be carefully read by every thoughtful dentist. There is also an excellent article by Dr. Geo. H. Cushing, upon "The Germ Theory in its Relation to Daily Practice." There are some rather meagre reports of societies, with correspondence, news-items, etc. The editorials are of quite a slashing character, and give evidence of a fearless if not a reckless hand at the helm. Altogether, the number is a good one, and if the same standard is adhered to *The Review* will not probably lack support.

TRANSACTIONS OF THE TEXAS STATE MEDICAL ASSOCIATION. Eighteenth Annual Session, held at Dallas, Texas, April, 1886.

This is a ponderous volume of nearly 700 pages, handsomely bound and printed. It is one of the most complete volumes of the kind that we have seen, and in the appearance of its transactions and the fullness of the reports, the Texas State Medical Society holds an easy lead. Much of the credit for such a beautiful volume is undoubtedly due to Dr. F. E. Daniel, the secretary of the society, and the editor and publisher of *Daniel's Medical Journal.*

THE PHYSICIAN'S VISITING LIST FOR 1887. Philadelphia: P. Blakiston, Son & Co.

This is the thirty-sixth year of the publication of this little volume. Besides being a visiting list, it contains a mass of information concerning doses, poisons, remedies, etc., that make it of great value to every one who practices in any field of medicine.

*First Annual Report of the State Board of Health and Vital Statistics of Pennsylvania for the year* 1885.

*On the Transplantation of the Eye of a Rabbit.* By LUCIEN HOWE, M. D.

*The Surgery of the Pancreas as Based upon Experiments and Clinical Researches.* By. N. SENN, M. D. Reprinted from the Transactions of the American Surgical Association.

*Surgical Notes from the Case Book of a General Practitioner.* By WILLIAM C. WILE, M. D. Reprinted from the *New England Medical Monthly.*

*Inter-State Notification;* as demonstrated in the history of Yellow Fever, at Biloxi, Miss.

*Address on Relation of Quarantine to Shipping Interests.* By JOSEPH HOLT, M. D.

*The Recording of Cases.* By LUCIEN HOWE, M. D.

*Four Cases of Extra-Uterine Pregnancy.* By MATTHEW D. MANN, M. D. Reprinted from *The Medical News.*

*Method in Medical Study.* By CHARLES H. MAY, M. D. Reprinted from *The New York Medical Journal.*

*Dr. Thomas Taylor's Reply to "Science."* Relating to the Crystals of Butter, Animal Fats and Oleomargarine.

Notices of other books and pamphlets are unavoidably postponed till another number.

